# Freshman 15 in England: a longitudinal evaluation of first year university student’s weight change

**DOI:** 10.1186/s40608-016-0125-1

**Published:** 2016-11-03

**Authors:** Claudia Vadeboncoeur, Charlie Foster, Nick Townsend

**Affiliations:** British Heart Foundation Centre on Population Approaches for Non-Communicable Disease Prevention, Nuffield Department of Population Health, University of Oxford, Old Road Campus, Oxford, OX3 7LF UK

**Keywords:** Obesity, Student, University, Weight gain, Freshmen 15

## Abstract

**Background:**

Weight change in first year university students, often referred to as ‘Freshman 15’, has been shown to be a common problem in North America. Studies have reported weight gain to be between 1 kg and 4 kg over the academic year and a recent meta-analysis found a mean gain of 1.34 kg and that 61 % of students gained weight. A limited number of studies have investigated weight change in England and large scale studies are needed to understand better weight change trends and to conduct subgroup analyses. This is important in the context of rising obesity prevalence, especially as behaviours and unhealthy weight in early adulthood often remains over the lifetime.

**Methods:**

We recruited students across 23 universities in England to complete a web-based survey at three time points in 2014–2015: beginning of academic year, December, end of academic year. Students were asked to self-report height and weight. We calculated weight change of each student between time points and conducted t-tests and pared analysis of variance to investigate the effect of time, sex and initial BMI. We also investigated weight change amongst weight gainers and in weight losers separately.

**Results:**

We followed 215 students over three time points and found a mean weight change of 0.98 kg (95%CI 0.49–1.47) over a mean length of 34 weeks of follow-up. The weight change rate was not significantly different over different terms. Over 51 % of the sample gained more than 0.5 kg by the end of the academic year, with a mean gain of 3.46 kg. Female weight gainers had a significantly lower baseline weight than non-weight gaining females. Twenty-five percent of the sample lost more than 0.5 kg with a mean of −3.21 kg. Within weight losers, males lost significantly more weight than females.

**Conclusion:**

Our findings reinforce that the first year of university is a crucial time in the life of students during which the majority tend to gain weight. However, we also found that 25 % lost weight, indicating that 75 % of students undergo a meaningful weight change in their first year. Universities must recognise their role in promoting healthy weight maintenance.

## Background

Obesity has increased significantly since 1980 [1] with global prevalence doubling in the past three decades [2]. The WHO reports that in 2014, 1.9 billion adults worldwide were obese or overweight [2]. The epidemic is thought to be costing $2 trillion globally every year [3] as it notably increases the incidence of diabetes, cancer and endocrine diseases [4]. Research since the 1980s has reported that students gain a significant amount of weight during their first year of university. The phenomenon has been called the ‘Freshman 15’, in reference to the perception that students gain 15lbs (6.8 kg) during their first year in university [5]. However, a mix of cross-sectional, cohort and randomised studies have shown weight change to actually be between −0.6 kg and 4 kg [6–35] with no consensus on differences between sexes in weight change over this time [36]. The literature on university student weight change shows that weight gain is linked to poor eating habits, stress and lower physical activity following the transition from a structured secondary education environment to university [37, 38]. Indeed, for first year university students, at 17–19 years old, it is often the first time they live away from home, have greater access to alcohol and are in an environment with many catering facilities [36]. Meta-analyses published on the topic between 2008 and 2015 have slightly varying estimates with pooled mean weight change of first year undergraduates found to be between 1.36 and 1.75 kg [36, 38, 39]. The most recent meta-analysis, from 2015, further investigated weight trends and found that more than 60 % of students gained weight and that weight gainers gained on average 3.38 kg (7.5lbs) (95%CI: 2.85–3.92) over the first academic year, supporting the argument that weight change should be considered beyond overall population means. More specifically, studies should perform subgroup analyses within weight gainers and weight losers and should investigate weight change as a rate (kg/week) to account for variability in follow-up lengths, to increase comparability between terms/semesters and to ensure standardisation across studies.

Unlike the US and Canada, the English (UK) context of student weight change over the first year of university has not been thoroughly investigated. In England, approximately 50 % of the youth (17–30 years old) attend higher education [40] and according to the National Union of Student, 20 % of students live at home during their undergraduate studies [41]. In contrast to the US, students can usually drink from their first year as the legal age is 18 years. In the most recent meta-analysis, only one longitudinal study on the subject was identified where Finlayson et al., found a weight change of 0.83 kg over three months in 120 students [42]. However, this study did not explore weight change during the second academic term nor analyse weight change in weight gainers and losers. It is worth further investigating students in England to extend our understanding of weight change in university students, especially as we are seeing increases in childhood and adult obesity in the country [43]. Evidence indicates that adolescent weight gain is highly linked to overweight and obesity in adulthood [44] and poor life habits gained during university may persist throughout adulthood. Particularly, it is important to explore weight change in relation to baseline weight, investigate weight change in rates and to conduct subgroup analyses amongst susceptible groups such as weight gainers and weight losers. Understanding weight change better at a large scale in England, in many universities, can provide insights as to the extent of the problems and shed light into possible interventions.

To answer these gaps on weight change in first year undergraduates in England, we aimed at sampling students across all universities in England. After data cleaning for eligibility, we gathered longitudinal data from students in 23 universities in England and obtained estimates on the mean weight change, the percentage of students who gained/lost weight and the amount of weight change stratified by these categories. We further tested the hypotheses of differences based on sex, academic terms and baseline BMI. For the first time, we also examined weight gainers and weight losers within baseline BMI categories and conducted analyses on individual relative weight change within these subgroups. Following recommendations from the 2015 meta-analysis to increase the quality of studies on the Freshman 15 [36], we analysed our data as a complete case approach, longitudinally and reported all essential data with standard deviations, including weight change in rate.

## Methods

### Participants

The study population was full time first year undergraduates studying in universities in England. As we were interested in the transition from secondary education to university, students who took a gap year or were older than 19 at the start of the academic year, were excluded. Students were also excluded if they reported never weighing themselves, were pregnant or had children and if they reported taking medication which may have affected their weight.

### Study design

Our study design was a longitudinal cohort with three time points, using the Online Bristol Survey as the web platform. We designed and pilot tested the survey which notably collected data on sex, age, ethnicity and self-reported weight and height. We obtained ethics approval from the University of Oxford (SSD/CUREC1A/14-093) and we contacted research ethics committees of all the other 100 universities from the England University League Table. We were subsequently able to recruit students from 28 universities across England. We could not recruit from all 101 universities due to procedural and content inconsistencies between research ethics committees across England [45]. Through the registrar, departments and student unions, we advertised our online survey at the beginning of the 2014 academic year. Students were asked to follow a link and complete the short, 10–15 min survey. All participants provided online consent and were entered in a draw for retail vouchers to encourage participation.

### Data collection

We collected data online over two weeks at each time point, T1 (start of university), T2 (before Christmas) and T3 (end of academic year). We compiled a list of university start dates and end dates from university websites to account for differences in academic calendars. Baseline (T1) data from students were collected within two weeks of the start of their university in September/October. T2 data were collected across all students at the beginning of December. T3 student data was obtained within two weeks of the end of their last teaching term. Only students who had completed T1 could participate in the follow-up surveys. Students provided their self-reported weight in kilograms (kg), pounds (lbs) or stone (st) and their height in metres (m) or feet (ft). From these, we calculated body mass index (BMI). We obtained weight change by subtracting, for each individual, self-reported weights collected at the different time points. We transformed all weight change into rates (kg per week) to account for variability in length of follow-up. Rates were obtained for each individual by dividing their weight change (kg) by their individual follow-up time (weeks) over the academic year and over each term.

### Data analysis

We used STATA 13.0 to calculate mean weight change over the different time points. We performed descriptive statistics, *t*-test and paired analysis of variance (ANOVA) to investigate the effect of time, sex, initial BMI and ethnicity. We also performed analyses stratified by weight gainers (students who gained more than 0.5 kg by the end of the academic year) and by weight losers (students who lost more than 0.5 kg over the academic year). The definition is based on a minimal threshold of weight to be gained as used in previous studies [10, 36]. Other studies on the topic have used any increase as a gain [27, 46] while others used a 5 % weight change from baseline weight [22]. Statistical significance was set at *p* < 0.05. We investigated total weight change, relative individual weight change as well as rate of weight change per week with a complete case approach.

## Results

### Recruitment and retention demographics

We recruited 1,126 participants for the T1 survey and of these, 599 completed T2 and 497 completed T3. Retention was 53 % from T1 to T2 and 44 % for T1 & T2 & T3. Since it was difficult to only target first year university students with online advertisement and due to restrictions imposed by universities, we had to exclude over 300 participants who were not in their first year. After screening for exclusion criteria, the final samples were 591 at T1, 311 at T2 and 279 at T3. Longitudinally, 311 students answered T1 and T2 while 215, from 23 universities, answered T1, T2 and T3 (Table 1).

**Table 1:** Descriptive demographics of the longitudinal student sample for those answering the first term only (*N* = 311) and those answering all time points (*N* = 215)

	First term only	All time points
Total n	311	215
Sex, n (%)		
Male	88 (28.3)	64 (29.7)
Female	223 (71.7)	151 (70.2)
Date of birth, n (%)		
1995	129 (41.5)	98 (45.6)
1996	178 (57.2)	115 (53.5)
1997	4 (1.3)	2 (0.92)
Origin, n (%)		
England	253 (83.5)	180 (86.5)
Wales/Scotland/NI	11 (3.6)	10 (4.8)
European Union	27 (8.9)	13 (6.3)
Outside EU	12 (4.0)	5 (2.4)
NA	7 (2.3)	7 (0.32)
Ethnicity, n (%)		
White	267 (85.9)	188 (87.4)
Mixed	7 (2.3)	4 (1.87)
Asian/Asian British	30 (9.7)	18 (8.41)
Black: African/Caribbean/British	4 (1.29)	2 (0.93)
Arab	3 (1.00)	3 (1.40)

The mean length of time between T1 and T2 (first term) was 9.4 weeks (range 7.5–11.5 weeks). Between T2 and T3 (second term), the mean number of weeks was 24 (range 19.5–25.4 weeks). For the whole academic year, between T1 and T3 (academic year), the mean length was 34 weeks (range 29–37 weeks). The T1-T2 sample was composed of 311 students; 72 % females and 28 % males. The majority of the sample reported being of white ethnicity and being from England (Table 1). The longitudinal sample, those who completed T1, T2 and T3 (215 students), was not significantly different than the first term only sample on mean baseline weight and sex composition. There were more females (70 %) than males (30 %) and 86.5 % reported being from England. The longitudinal sample of 215 students was used in all analyses presented here. In this sample, 73 % lived on campus. When investigating the type of housing arrangements, 8.4 % lived at home with parents, 20.0 % in catered university accommodation, 59.5 % in self-cratering university accommodation, 8.4 % in self-catering and catered university accommodation and 3.7 % in private house/flat with other students.

### Baseline results

At baseline, within two weeks of starting university, the mean weight of the males was 74.7 kg (SD = 17.9) and 60.2 kg (SD = 12.0) for females (Table 2). The mean height for males was 1.80 m (SD = 0.1) and 1.70 m (SD = 0.1) for females. Over 65 % of the males and females had a normal BMI while 10–12 % were underweight, 17.2 % of the males and 9.9 % of the females were overweight and 4.6 % of both males and females were obese.

**Table 2:** Baseline weight, height and BMI of males and females for the academic year longitudinal sample (*N* = 215)

	Male	Female
Total n (%)	64 (29.7 %)	151 (70.2 %)
Mean weight, kg (SD)	74.7 (17.9)	60.2 (12.0)
Mean height, m (SD)	1.8 (0.1)	1.7 (0.1)
BMI, n (%)		
Underweight	7 (10.6)	19 (12.5)
Normal	43 (67.2)	110 (73.0)
Overweight	11 (17.2)	15 (9.9)
Obese	3 (4.6)	7 (4.6)

### Weight change over the first year

Over the full academic year, there was a significant increase in weight in the sample by 0.98 kg (SE = 0.25, SD = 3.66) and a mean BMI increase of 0.42 (Table 3). We found no significant differences in mean weight change between males (+0.85 kg) and females (+1.04 kg). There was no significant difference in weight gain between those who were followed for less than the mean length of follow-up (34 weeks), compared to those followed for more than the mean. The mean weight change rate was +0.029 kg/week (SD = 0.10). The rate of weight change was +0.0051 kg/week for the first term, males lost weight at −0.0088 kg/week and females gained 0.11 kg/week. Although the difference between males and females was large for the first term, it was not significantly different. Over the second term, males gained on average 0.039 kg/week and females 0.037 kg/week. Despite the higher rate of weight gain for females and a weight loss amongst males during the first term, no significant difference in weight change rate was found between these periods.

**Table 3:** Mean weight, BMI and rate change over the academic year and different terms (*N* = 215)

	Number	Weight change (kg)	Weight change rate (kg/week)	BMI change
		Mean (SD)	Mean (SD)	Mean (SD)
Academic year	215	0.98 (3.66)	0.029 (0.10)	0.42 (1.49)
Male	64	0.85 (4.48)	0.025 (0.13)	0.39 (1.62)
Female	151	1.04 (3.27)	0.030 (0.09)	0.44 (1.44)
First term	215	0.06 (2.70)	0.005 (0.29)	0.06 (1.39)
Male	64	−0.13 (3.08)	−0.009 (0.33)	−0.04 (1.16)
Female	151	0.13 (2.54)	0.011 (0.27)	0.10 (1.48)
Second term	215	0.93 (3.56)	0.038 (0.15)	0.36 (1.45)
Male	64	0.98 (4.03)	0.039 (0.16)	0.99 (4.03)
Female	151	0.90 (3.35)	0.037 (0.14)	0.90 (3.35)

### Weight change in weight gainers

Over the full academic year, 52 % were classified as weight gainers (>0.5 kg gained over the academic year) with a weight gain range of 0.6–14.9 kg. The mean weight gain amongst weight gainers was 3.46 kg (SD = 2.57) (Table 4), compared to the overall sample mean of 0.98 kg (SD = 3.66). The mean weight gain rate in weight gainers was 0.10 kg/week. Over the whole academic year, male weight gainers gained statistically more weight, than female weight gainers (*p* < 0.01). However, there was no statistical difference in the change in weight relative to baseline weight (males = 6.1 %, females = 5.4 %). Twenty-seven students (12.5 %) gained at least 10 lbs (4.5 kg) and nine (4 %) gained the predicted 15 lbs (6.8 kg). Over T1-T2-T3, weight gainers gained on average 0.80 kg during the first term, at a rate of 0.083 kg/week, while they gained 2.66 kg over the rest of the academic year at a similar rate of 0.11 kg/week. The rate of weight gain was not significantly higher between the first term compared to the second term.

**Table 4:** Mean weight change and rate by period of time and sex, for those who gained and lost more than 0.5 kg by the end of the academic year (*N* = 165)

	Number	Weight change (kg)	Weight change rate (kg/week)
		Mean (SD)	Mean (SD)
Academic year			
Weight gainers	111	3.46 (2.57)	0.10 (0.07)
Male	30	4.33 (2.83)	0.12 (0.08)
Female	81	3.13 (2.41)	0.09 (0.07)
Weight losers	54	−3.21 (2.94)	−0.09 (0.08)
Male	17	−4.43 (3.36)	−0.13 (0.09)
Female	37	−2.65 (2.59)	−0.08 (0.07)
First term			
Weight gainers	111	0.80 (2.34)	0.08 (0.26)
Male	30	0.98 (2.60)	0.11 (0.27)
Female	81	0.73 (2.26)	0.07 (0.25)
Weight losers	54	−1.19 (3.09)	−0.13 (0.31)
Male	17	−1.70 (3.70)	−0.17 (0.38)
Female	37	−0.96 (2.81)	−0.11 (0.28)
Second term			
Weight gainers	111	2.66 (3.15)	0.11 (0.13)
Male	30	3.35 (3.55)	0.13 (0.14)
Female	81	2.40 (2.97)	0.10 (0.12)
Weight losers	54	−2.02 (3.04)	−0.08 (0.12)
Male	17	−2.74 (2.96)	−0.11 (0.12)
Female	37	−1.69 (3.06)	−0.07 (0.13)

Those who gained over 0.5 kg during the first term, using 0.5 kg as an indication for early weight gainers, gained an average of 2.42 kg over the first term at a rate of 0.26 kg/week (Table 5). These same individuals continued to have an academic year average weight gain of 2.33 kg at a rate of 0.07 kg/week. While for the second term, those who gained at least 0.5 k during that period, gained 3.50 kg over the second term at a rate of 0.14 kg/week and ended the academic year with an overall weight change of 2.98 kg at a rate of 0.11 kg/week.

**Table 5:** Longitudinal mean weight change and rate, comparing the first term and second term weight change to final academic year weight change, for those who gained and lost weight over specific periods

		Period weight change >0.5 kg		Full year weight change	
	N	Weight change (kg)	Weight change rate (kg/week)	Weight change (kg)	Weight change rate (kg/week)
		Mean (SD)	Mean (SD)	Mean (SD)	Mean (SD)
		First term weight change > 0.5 kg		Academic year weight change	
First term					
Weight gainers	82	2.42 (1.83)	0.26 (0.20)	2.33 (3.32)	0.068 (0.10)
Male	24	2.61 (1.50)	0.29 (0.16)	2.35 (3.32)	0.068 (0.10)
Female	57	2.34 (1.95)	0.25 (0.21)	2.32 (3.35)	0.068 (0.01)
Weight losers	65	−2.84 (2.13)	−0.30 (0.22)	−0.63 (3.97)	−0.018 (0.11)
Male	21	−3.50 (2.43)	−0.37 (0.25)	−1.34 (4.91)	−0.037 (0.14)
Female	44	−2.52 (1.92)	−0.27 (0.20)	−0.30 (3.45)	−0.008 (0.01)
		Second term weight change > 0.5 kg		Academic year weight change	
Second term					
Weight gainers	105	3.50 (2.69)	0.14 (0.11)	2.98 (3.09)	0.085 (0.09)
Male	30	4.19 (2.06)	0.17 (0.12)	3.81 (3.37)	0.110 (0.09)
Female	74	3.22 (2.54)	0.13 (0.11)	2.65 (2.93)	0.076 (0.08)
Weight losers	56	−3.05 (2.44)	−0.13 (0.10)	−2.04 (3.65)	−0.058 (0.10)
Male	20	−3.19 (2.28)	−0.13 (0.10)	−2.99 (3.92)	−0.084 (0.11)
Female	36	−2.98 (2.55)	−0.12 (0.10)	−1.50 (3.44)	−0.043 (0.10)

Thirty students gained over 0.5 kg during the first term and during the second term (Table 6). For these individuals, the full year overall mean weight gain was 5.28 kg (SD = 2.93), significantly higher than the overall mean weight gain in general weight gainers. These students gained an average 2.18 kg for the first term and 3.10 kg for the second term, although the rate of weight change between periods was not significantly different.

**Table 6:** Mean weight change in weight gainers and weight losers at different periods for those who gained or lost more than 0.5 kg over the first term and second term (*N* = 40)

	Number	Weight change (kg) Mean (SD)		
		First term	Second term	Academic year
Weight gainers	30	2.18 (1.58)	3.10 (2.36)	5.28 (2.93)
Weight losers	10	−4.00 (2.94)	−2.68 (2.32)	−6.69 (4.56)

### Weight change in weight losers

Over the academic year, 25 % of students lost more than 0.5 kg with a mean weight loss of 3.21 kg (SD = 2.94) (Table 4) and a loss range of 14.5 kg to 0.7 kg. Males (−4.43 kg) on average lost significantly (*p* < 0.02) more weight than females (−2.65 kg) and on average lost significantly more relative baseline weight (5.8 %) than females (4.0 %). When investigating the full academic year, those who lost at least 0.5 kg over the academic year, −1.19 kg was lost during the first term, at a rate of −0.13 kg/week, while on average −2.02 kg was lost during the second term, with a rate of −0.083 kg/week (Table 4). The rate of weight loss was not significantly different between terms.

For the first term, those who lost over 0.5 kg during that period, using 0.5 kg as an indication for early weight losers, lost an average of 2.42 kg (Table 5). These students ended the academic year with a mean weight loss of 0.63 kg at an overall weight loss rate half of what it was during the first term. For the second term, those who lost at least 0.5 kg during that period, lost 3.05 kg at a rate of 0.13 kg/week. These same individual had an annual weight loss rate of 0.058 kg/week, significantly smaller than at the first term.

Ten students lost more than −0.5 kg over the first and second term to finish the academic year with an average weight loss of 6.69 kg over the 7–8 months (Table 6). For these students, the majority of the weight was lost during the first term.

### Weight change based on baseline weight and BMI

The baseline BMI category of students did not have a significant effect on the overall sample mean weight gain. The mean baseline weight of weight gainers did not significantly differ to the mean baseline weight of non-weight gainers. Although, female weight gainers had a significantly lower mean baseline weight (58.4 kg) than female non-weight gainer (62.1 kg). There was no significant differences in males with regards to baseline weight in weight gainers (74.2 kg) and non-weight gainers (75.2 kg). Within the sub-sample of weight gainers, 15.3 % had a baseline BMI categorised as underweight, 72.1 % as normal, 8.1 % as overweight and 4.5 % as obese (Table 7). Weight gainers in the underweight and obese BMI categories did not gain a greater mean relative weight change compared to weight gainers in the normal BMI category.

**Table 7:** Baseline BMI and mean relative weight change in weight gainers and weight losers based on baseline BMI categories (*N* = 165)

Category	Number	Percent	Mean BMI (SD)	Mean relative (%) weight change (SD)
Weight gainers				
Overall	111			
Underweight	17	15.32	17.25 (0.75)	6.90 (3.40)
Normal	80	72.07	21.47 (1.78)	5.36 (4.75)
Overweight	9	8.11	27.16 (1.48)	4.96 (3.75)
Obese	5	4.50	35.85 (3.21)	6.81 (3.99)
Weight losers				
Overall	54			
Underweight	4	7.41	17.41 (0.30)	−3.10 (1.54)
Normal	37	68.52	21.50 (1.85)	−4.44 (2.95)
Overweight	11	20.37	27.00 (1.37)	−4.89 (3.99)
Obese	2	3.70	34.22 (4.40)	−8.43 (8.17)

The mean baseline weight of weight losers was not significantly different to the mean baseline weight of non-weight losers. When stratified by sex, female weight losers had a significantly higher mean baseline weight (64.0 kg) compared to female non-weight losers (58.9 kg). Male weight losers (72.3 kg) did not have significantly different baseline weight than non-losers (75.5 kg), although the difference was large. From the 54 weight losers, 7.4 % had a baseline BMI in the underweight category, 68.5 % in the normal category, 20.3 % in the overweight and 3.7 % in the obese category. Weight losers in the underweight and overweight BMI categories did not lose a greater mean relative weight change compared to weight losers in the normal category.

## Discussion

In our study, the overall mean weight change over 7–8 months was 0.98 kg (95 % CI 0.49–1.47) at a rate of 0.029 kg/week (SD = 0.10). There was no difference by sex or in the rate by term. We found that 12.5 % of the sample gained over 4.5 kg and that 52 % of the sample gained over 0.5 kg by the end of the academic year. The average weight gain in weight gainers was 3.46 kg (95%CI: 2.98–3.94). Within weight gainers, males gained significantly more weight and significantly more relative weight than females (*p* < 0.05). Female weight gainers had significantly lower baseline weight than female non-weight gainers. Thirty students gained over 0.5 kg during both the first and second term and finished the academic year with a large weight gain of 5.28 kg (SD = 2.93). From our sample, 25 % of the students lost weight with an average of −3.21 kg, with males losing significantly more relative weight and at a significantly faster rate than females.

One of the main strengths of this study was its longitudinal design across several universities in England. The final longitudinal sample was composed of 215 students from 23 different universities, in all regions of England, representing 0.2 % of the first year students in the sampled universities. According to a recent meta-analysis [36], this study would be the largest longitudinal study in England and the sixth largest in the world on the topic. The data we collected allowed us to stratify mean weight gain by sex, weight gainers and weight losers to better understand the Freshmen 15. By calculating weight change per week, we were able to investigate the rate of weight change over the academic year. We also analysed data as complete case and for the first time, presented relative weight change and relative weight change by BMI category. Some of the limitations of this study include the use of varied follow-up lengths, self-reported weight and below 50 % retention rate. The varied follow-up length is due to the nature of the English university system with unequal terms and academic years. We accounted for this by using rates of weight change. We tested for the impact of different follow-up lengths and found no significant effect. With regards to self-reported weight, there is a tendency to under-report weight and over-report height [47], though the possible bias has been shown to be minimal and consistent across time points in the student population [48, 49]. Since we are looking at weight change from longitudinally reported weights, the impact of self-report is reduced. Two meta-analyses showed no significant difference between mean weight change in studies when comparing studies that had measured weight and self-reported weight [36, 39]. One of the meta-analyses also found no significant effect of low, medium and high retention rates [36]. Our study was longer than the average of 5 months for studies on the topic and had a slightly lower retention rate than the average of 57 %. Nevertheless, we acknowledge there may be some reporting bias and selection bias, notably healthy responder bias, leading to misclassification and potentially conservative estimates. Another limitation of this study is that it is a descriptive study as we have no control groups of non-university students. Furthermore, we had wished to perform multi-level analyses of students nested within universities but the sample per university was not large enough. Overall, our study managed to account for recommendations of past meta-analyses by reporting the percentage of weight gainers/losers, stratifying analyses by these subgroups, reporting standard deviations and analysing from a longitudinal complete-case perspective.

This study further supports evidence that students in their first year of university gain a significant amount of weight and at a faster rate than the general population. The weight gain found in a 5 year follow-up study of approximately 20,000 health-conscious UK residents was 0.4 kg/year [50]. Our overall student sample mean weight gain over the academic year was within the range of similar studies published on this topic. It is statistically similar to results from a 2015 meta-analysis which found a pooled mean of 1.36 kg (95%CI 1.15–1.57) [36]. Similarly to the majority of comparable studies, we did not find a statistical difference based on sex for the overall mean [36]. In our study, this is likely due to lack of power and the imbalance in the male – female ratio (30:70). Our surveyed population of first year university students comprised 16.7 % of overweight or obese at baseline, lower than the country average of 30 to 36 % for 16–24 year old in England [51]. This is likely due to our sample being purely university students, being of 17 and 18 years of age along with potential volunteer bias.

We found a comparable proportion of weight gainers to the pooled estimate of 60.7 % from the meta-analysis. Across the literature, the range in percentage of weight gainers is between 22 % and 93 %, notably studies do not have the same definition of weight gainers [36]. The mean weight gain in weight gainers was statistically similar to the 3.38 kg (95%CI: 2.84–3.92) found in the meta-analysis of five studies [36]. This weight change in weight gainers is 4.6 times faster than the average weight gain in Scottish young adults (20–25 years old) [52] and 3.5 times the 1 kg average annual weight change in young adults in the US, according to the CARDIA study which followed adults for 14 years [53]. We found that 25 % of our sample lost weight, similar to a weighted average of 22 % from a range of 6 to 31 % found in studies identified by the meta-analysis [36]. The mean weight loss in weight losers of −3.21 kg was similar to the mean found by another study [27]. We are the first study to thoroughly investigate weight change in weight gainers and weight losers in rates and to explore relative weight change within subgroups.

As discussed in the meta-analysis, interpreting the overall mean results of a large population can be misleading and neglect the aspect that some individuals gain a lot while some lose a lot. It was 75 % of our sample that had a greater weight change than 0.5 kg. Within both weight gainers and weight losers, it seems apparent that there is an important and meaningful weight change of 3.46 kg (5.6 % of baseline weight) and −3.21 kg (4.6 % of baseline weight) respectively, at rates of 0.10 kg/week. Within the discussion of the Freshmen 15 which usually has a weight gain association, we must recognise that within this population, a large number of first year students lose weight, with a high averaged weight lost over the academic year. Interestingly, we found that for many individuals, a large weight loss during the first term did not necessarily mean a large academic year weight loss. Similarly, some students who had a large first term weight gain had small weight loss during the second term. This suggests that it is crucial to better understand the trajectory of this weight increase/decrease to attenuate this trend and prevent health consequences. Previous authors have identified physical inactivity, poor eating habits, stress and baseline weight linked to weight gain in the overall sample [37, 38]. Within weight gainers/losers, these influencers may have an exacerbated effect or other influencers pay a key role.

## Conclusion

Our study is an important further steps into providing greater knowledge on the weight change trends in first year students. Students do experience important weight changes and universities need to focus on increasing health promotion to help them maintain/adopt healthy behaviours. Beyond this, it is important to understand the individual, interpersonal and environmental factors which influence weight gainers and weight losers, especially in the English context. Future studies should aim for large sample sizes to allow stratification and identification of significant correlates and studies should span more than one year to track trends following the first year of university. A study adopting the socio-ecological framework would allow for a holistic understanding of weight change in students.

## Abbreviation

BMI: Body mass index
CI: Confidence interval
