# Erratum to: Freshman 15 in England: a longitudinal evaluation of first year university student’s weight change

**DOI:** 10.1186/s40608-016-0132-2

**Published:** 2016-12-09

**Authors:** Claudia Vadeboncoeur, Charlie Foster, Nick Townsend

**Affiliations:** British Heart Foundation Centre on Population Approaches for Non-Communicable Disease Prevention, Nuffield Department of Population Health, University of Oxford, Old Road Campus, Oxford, OX3 7LF UK

## Erratum

Following publication of the original article [1] it was brought to our attention that one of the authors’ funders had been omitted from the acknowledgements. Please therefore note that the revised acknowledgements section should read as follows:

“N.T. (006/P&C/CORE/2013/OXFSTATS), and C.F. (006/PSS/CORE/2016/OXFORD) receive funding from the British Heart Foundation (BHF). CV is grateful for Clarendon and Nuffield Department of Population Health funding.”
